# Comparison of Methods for Factor Invariance Testing of a 1-Factor Model With Small Samples and Skewed Latent Traits

**DOI:** 10.3389/fpsyg.2018.00332

**Published:** 2018-03-22

**Authors:** Holmes W. Finch, Brian F. French, Maria E. Hernández Finch

**Affiliations:** ^1^Department of Educational Psychology, Ball State University, Muncie, IN, United States; ^2^Learning and Performance Research Center, Washington State University, Pullman, WA, United States

**Keywords:** invariance, factor analysis, validity, small samples, estimation, Bayesian

## Abstract

A primary underlying assumption for researchers using a psychological scale is that scores are comparable across individuals from different subgroups within the population. In the absence of invariance, the validity of these scores for inferences about individuals may be questionable. Factor invariance testing refers to the methodological approach to assessing whether specific factor model parameters are indeed equivalent across groups. Though much research has investigated the performance of several techniques for assessing invariance, very little work has examined how methods perform under small sample size, and non-normally distributed latent trait conditions. Therefore, the purpose of this simulation study was to compare invariance assessment Type I error and power rates between (a) the normal based maximum likelihood estimator, (b) a skewed-*t* distribution maximum likelihood estimator, (c) Bayesian estimation, and (d) the generalized structured component analysis model. The study focused on a 1-factor model. Results of the study demonstrated that the maximum likelihood estimator was robust to violations of normality of the latent trait, and that the Bayesian and generalized component models may be useful in particular situations. Implications of these findings for research and practice are discussed.

## Introduction

The field of psychology relies heavily on the use of tools for measuring constructs as varied as cognition, mood, personality, and attitude. Scores derived from these instruments are frequently used to assist in making decisions about whether a child should receive special educational accommodations, whether a patient may be suffering from depression, what type of employment an applicant may best be suited for, and whether a college student is motivated by an internal or external reward structure, among others. The ubiquity of such tools, coupled with the importance of their use in decision making means that it is crucial that they provide comparable information for individuals from across the population (American Educational Research Association et al., 2014). For example, a school psychologist using a measure of social functioning for children with Autism depends on the comparability of scores for boys and girls. If this assumption is not tenable, then decisions based on scores derived from the scale may not have the same meaning for individuals from the two genders, potentially leading to improper provision (or lack thereof) for some people (Millsap, 2011). As a result, it is crucial that practitioners and researchers know whether the items on the scale are invariant or the same across theoretically interesting subgroups. In this context, invariance refers to the case when individual items have the same statistical characteristics (e.g., relationship to the latent trait being measured by the scale) for members of the various subgroups (e.g., boys and girls). Such investigations help address a component of a fairness argument (Dorans, 2004).

Given the importance of scales in the social sciences, and the need to assess whether they are, in fact, invariant across subgroups in the population, a variety of statistical tools have been developed for this purpose (Osterlind and Everson, 2009; Millsap, 2011). Perhaps the most common approach for invariance testing is based upon the multiple groups confirmatory factor analysis (MGCFA) model (e.g., Byrne et al., 1989; Bollen, 2009), which is described below. This approach involves the fitting of two CFA models to a set of data. One of these models constrains factor model parameters to be equal, whereas the other allows some of the parameters to vary between groups. The statistical fit of the models are compared using the difference of some index, such as the chi-square goodness of fit statistic, or the comparative fit index, or the combination of the two (e.g., Cheung and Rensvold, 2002; French and Finch, 2006). If the fit of the models is found to differ, the conclusion is reached that one or more of the unconstrained parameters differs between the groups of interest (e.g., Byrne et al., 1989). Much of the researcher investigating the performance of invariance testing methods has focused on cases in which the group sizes are moderate to large (100 or more), and where the latent trait is normally distributed. However, in practice researchers may be faced with small samples, such as when working with individuals who have been identified with Autism, or some low incidence population that may pose unique sampling challenges. In addition, the latent trait of interest may not be normally distributed. Indeed, variables such as socio-economic status, depression, anxiety, academic motivation are likely skewed in the population. For example, in the general population most individuals may have relatively low scores on a depression inventory, but a few individuals will have higher scores, or clinically significant scores (i.e., more depression), leading to a positively skewed distribution.

Little work has documented model invariance testing method accuracy for small sample conditions and/or skewed latent trait distributions. Thus, the goal of the current study is to add to the literature by considering several approaches for invariance assessment in such circumstances. The remainder of this manuscript is organized as follows: First, the common factor model is described, and the standard MGCFA approach to invariance testing briefly reviewed. Next, alternative methods of assessing invariance are reviewed, including approaches based on the skewed-*t* distribution, a components based model, and Bayesian estimation. The goals of the study and a description of the simulation study designed to address these goals appear next. Finally, results of the simulation study, as well as a brief illustration using an extant dataset, and then a discussion of the results are presented.

### Common factor model and invariance

At the heart of most psychological measures lies a construct, such as motivation, anxiety, or reading ability. Such constructs are assumed to be the primary cause of responses to items on the scale assessing that construct, in addition to random variability that is unrelated to the construct. We can express the relationship between these observed indicators (i.e., the items) and the latent trait measured using the common factor model, which takes the following form (Bollen, 2009):

(1) $$\begin{array}{r} x=\tau +\Lambda \xi +\delta \end{array}$$

Where

*x* = Vector of observed indicator variables;e.g.items on ascale or subscale scores

ξ = Vector of latent traits being measures by *x*

Λ = Matrix of factor loadings linking *x* and ξ

τ = Vector of intercepts associated with *x*

δ = Vector of unique errors associated with *x*

The model in (1) implies the following covariance matrix for the observed indicators:

(2) $$\begin{array}{c} \Sigma =\Lambda \Psi \Lambda^{\prime }+\Theta \end{array}$$

Where

Σ = Covariance matrix of the observed indicators, *x*

Ψ = Covariance matrix of the latent factors

Θ = Covariance matrix of unique error terms, assumed to be diagonal.

Parameter estimation in Equations (1) and (2) can be completed via a variety of methods, with perhaps the most common being maximum likelihood (ML). ML is designed to find the model parameter estimates (e.g., loadings, intercepts) that optimize the following function:

(3) $$\begin{array}{r} F_{ML}=ln|S|-ln|\Sigma |+trace\left[S\Sigma^{-1}\right]-p \end{array}$$

Where

*S* = Observed covariance matrix for the indicator variables

Σ = Model implied covariance matrix for the indicator variables

*p* = Number of observed indicators.

Thus, ML identifies the parameter estimates that result in the smallest possible difference between the actual covariance matrix among the indicators, and the model predicted covariance matrix. ML is an iterative process, which repeatedly obtains and updates parameter estimates, until model convergence is achieved. This is signaled when the value in Equation (3) falls below a predetermined cutoff value (e.g., 0.000001). ML relies on an assumption that the observed indicators follow a normal distribution (Kline, 2016).

### Multiple group confirmatory factor analysis for invariance assessment

Factor invariance (FI) is an assumption underlying the use of the common factor model in Equation (1). Simply stated, the presence of FI means that the parameters in Equations (1) and (2) are equal across two or more subgroups within the population (Millsap, 2011). Such subgroups might include males and females, individuals with a specific medical diagnosis and those without such a diagnosis, or those in different age cohorts. Under the broad heading of FI, there exist subtypes of factor model invariance that are differentiated by invariance of specific factor model parameters. The weakest type of FI is known as configural invariance (CI), which rests on the assumption that the number of latent variables, and the correspondence of observed indicators to these latent variables is the same for all groups in the population, but that the values of the parameters themselves might differ (see Millsap). If CI is present, researchers typically next investigate metric invariance (MI), which assumes that the factor loadings (Λ) are equivalent across groups, implying that the latent variables are being measured in the same way for members of the population subgroups under consideration (Wicherts and Dolan, 2010; Kline, 2016). Said another way, the relationship between the variable and the factor are equal across groups. MI assessment is the focus of this study. If MI holds, researchers next assess equality of the factor model intercepts (τ) across groups, as well as that of the unique indicator variances (δ). This last type of invariance, known as strict factor invariance (SFI; Meredith, 1993) thus implies that the factor loadings, intercepts, and unique variances are all equivalent across groups. Millsap (2011) indicated that SFI is necessary in order to attribute group differences in the mean and covariance structure of the observed indicators to corresponding differences at the latent variable level.

Perhaps the most common methodology for assessing FI involves the use of a multiple groups CFA (MGCFA) model:

(4) $$\begin{array}{r} x_{g}=\tau_{g}+\Lambda_{g}\xi +\delta_{g} \end{array}$$

The terms in Equation (4) are exactly as described in Equation (1), with the addition of the *g* subscript, indicating that parameters can vary by group. Likewise, the covariance matrix in Equation (2) is written in the MGCFA context as:

(5) $$\begin{array}{c} \Sigma_{g}=\Lambda_{g}\Psi_{g}{\Lambda^{\prime }}_{g}+\Theta_{g} \end{array}$$

such that groups are allowed unique observed covariance matrices (Σ_*g*_), factor loadings (Λ_*g*_), factor covariance matrices (Ψ_*g*_), and unique error matrices (Θ_*g*_).

MGCFA can be used to test each aspect of FI using a series of nested models in which specific parameters are constrained to be equal across groups, and then allowed to vary by group. For example, in order to assess MI, a model is fit to both groups in which the factor loadings (as well as the intercepts and error variances) are constrained to be equal across groups. A second model is then fit in which the loadings are allowed to differ across groups, but the intercepts and error variances remain constrained to be equal. The fit of the two models, which are nested, can then be compared using a difference in chi-square goodness of fit statistic. This difference in model χ^2^ values, which appears in Equation (6), follows a χ^2^ distribution.

(6) $$\begin{array}{r} \chi_{\Delta }^{2}=\chi_{loadings\;constrained}^{2}-\chi_{loadings\;unconstrained}^{2} \end{array}$$

Where

$\chi_{loadings\;constrained}^{2}$ = chi - square fit statistic for the model with constrained loadings

$\chi_{loadings\;unconstrained}^{2}$ = chi - square fit statistic for the model with unconstrained loadings

The $\chi_{\Delta }^{2}$ statistic assesses the null hypothesis that the fit of the two models is equivalent; i.e., the groups have equal factor loadings in the population. If the $\chi_{\Delta }^{2}$ test is statistically significant at a predetermined level of α (e.g., 0.05), the researcher would conclude that there is not MI in the population. This same technique can be applied to other fit indices (CFI) to assess change in fit (e.g., Cheung and Rensvold, 2002) or can be used in combination with the chi-square difference test.

### Maximum likelihood based on the skewed-*t* distribution

As noted above, the ML algorithm used in CFA parameter estimation is based upon the assumption that the observed indicator variables are normally distributed. However, in some instances this may not be a valid assumption about the data. For example, observed indicator scores, as well as the latent traits of some psychological or educational phenomena may yield a skewed distribution, such as on social interaction scores for individuals with Autism. In such cases, models based on the standard ML approach may produce biased parameter estimates (Lin et al., 2013; Asparouhov and Muthén, 2016). In turn, such parameter estimation bias could negatively influence the results of invariance testing. We note that skewness in observed indicators could be a function of skewness in the latent trait, as well as skewness in the individual indicator variables themselves. The focus of this study is on skewness that originates only at the latent trait level.

As a means of addressing skewness in the data, Asparouhov and Muthén (2016) describe the use of a restricted skewed-*t* ML estimator in the context of factor analysis, structural equation modeling, and mixture modeling parameter estimation. They note that the skewed-*t* subsumes a number of other distributions, including the skewed-normal, making it a suitably general approach for use in a wide array of circumstances. We do not provide technical details of ML based on the skewed-*t*, but would refer the interested reader to the very readable paper by Asparouhov and Muthén. Nonetheless, a few technical points with regard to the skewed-*t* do need to be addressed here. First, the estimation algorithm assumes that a set of variables comes from the restricted multivariate skewed-*t* distribution, which is characterized by 4 sets of parameters:

μ = Vector of means

Σ = Covariance matrix

δ = Vector of skewness parameters

ν = Degrees of freedom parameter.

The skewed-*t* distribution has *P*+1 more parameters to be estimated than is the case for the normal distribution, where *P* is the number of observed indicators. These additional parameters include the skewness values for each indicator, and the single degrees of freedom parameter for the model. In short, the estimation burden placed on the data by the skewed-*t* estimator is greater than that of the normal based approach. These parameters are then estimated using an iterative ML approach with the goal of minimizing the difference between the observed and model implied covariance matrices, much as with the standard ML in Equation (3), though the optimization function is different. Readers who are interested in the derivation and form of this optimization function are referred to Lin et al. (2013). Invariance testing based on the skewed-*t* distribution follows closely with that described above for the ML assuming a normal distribution.

### Generalized structured components analysis

Generalized Structured Components Analysis (GSCA) is a latent variable modeling methodology that defines one or more composite variables from a set of observed indicators. As described by Hwang and Takane (2004), GSCA defines a latent variable as a weighted linear composite of the indicators in a manner very similar to that used by principal components analysis (PCA). In addition, GSCA can also be viewed as a close relative of partial least squares (PLS) modeling. However, GSCA has a global optimization function, which is not true for PLS, potentially yielding more stable and in some cases less biased estimates (Hwang and Takane, 2004). The latent variable in GSCA is expressed as a weighted composite of a set of observed indicator variables:

(7) $$\begin{array}{r} \gamma =wz \end{array}$$

Where

*z* = data matrix of form *N*x*J* (observations by indicators)

*w* = *J*x*T* matrix of measurement weights (indicators by composite variables)

The GSCA model can then be written as:

(8) $$\begin{array}{r} z=a\gamma +\varepsilon \end{array}$$

Where

*a* = Component loadings

ε = Model error

The right side of Equation (8) contains predicted values of the indicators as a function of the latent component (*aγ*), as well as an estimate of the error associated with the indicators (*e*). Hwang and Takane (2010) found that this estimate of model error does not completely adjust the component estimate for random measurement error, but that the inclusion of more indicator variables does result in the proportion of observed indicator variance that is associated with this error. In other words, GSCA approximates the factor model using a weighted composite and the component loadings linking it to the observed indicators, but does not estimate it directly, as is the case with the other methods included in this study. The resulting model parameter estimates (e.g., component loadings) are therefore more accurate when more indicators are included in the model. The fitting function for GSCA is:

(9) $$\begin{array}{r} f_{GSCA}=SS\left(z-a\gamma \right) \end{array}$$

Equation (9) reflects the fact that the GSCA parameter estimates are found to minimize the sum of squares between values of the observed indicator variables, and the predicted indicator values based on the latent component value and loadings. Hwang and Takane (2004) describe an alternating least squares algorithm for estimating the model parameters, *a* and *w*. In the first step, *w* is fixed (initially at 1, but then allowed to vary in subsequent steps) and *a* is estimated. In step 2, the estimates of *a* from step 1 are taken as fixed and values of *w* are estimated. These steps are repeated until convergence of the fitting function is achieved. An important point to note here, particularly in light of the goals of this study, is that no distributional assumption are made for either *z* or γ in the estimation of GSCA parameters *a* and *w*.

As with any statistical modeling, an important issue when using GSCA is to determine the extent to which the model fits the observed data. Several statistics have been proposed for this purpose, with perhaps the strongest candidate being the adjusted FIT (AFIT) index (Hwang et al., 2007). This value is calculated as:

(10) $$\begin{array}{r} AFIT=1-\left(1-\frac{1}{T}\left(\overset{T}{\underset{t=1}{\sum }}R_{t}^{2}\right)\frac{d_{0}}{d_{1}}\right) \end{array}$$

Where

$R_{t}^{2}\;=$ Variance explained in indicator variable *t* by the latent variable structure.

*d*_0_ = Degrees of freedom for the null model (*NJ*).

*d*_1_ = Degrees of freedom for the model being fit to the data (*NJ-G*).

*G* = Number of free parameters.

*AFIT* expresses the proportion of variability in the observed variables by the latent model expressed in Equation (8), adjusted for model complexity. AFIT ranges between 0 and 1, with larger values indicating better model fit to the data.

Given the GSCA model formulation expressed above, invariance of parameters across groups can be tested by fitting two models, and then comparing their fit, much as was the case with MGCFA, described earlier. The first of the models would constrain the model parameters (e.g., component loadings) to be equal across groups, whereas the second model would allow one or more of these parameters to differ among groups. Model invariance can then be tested by comparing the AFIT values of the two models using a dependent samples *t*-test (Hwang and Takane, 2015). The null hypothesis of this test is that the models fit the data equally well; i.e., *H*_0_ : *AFIT*_*constrained*_ = *AFIT*_*unconstrained*_. A statistically significant *t*-test would thus lead us to reject the null hypothesis, and conclude that at least one of the GSCA model parameters differs between groups. In the current study, the focus is on measurement invariance, and thus the unconstrained model will allow component loadings to differ between groups, whereas the constrained model will force or constrain them to be equal between the groups.

### Invariance testing using bayesian estimation

The final invariance testing approach we examined was based on the Bayeisan estimation paradigm. Muthén and Asparouhov (2012) described an approach to fitting structural equation models that used the Gibbs sampler in the context of markov chain monte carlo (MCMC) estimation. They demonstrated that MCMC estimation may be particularly useful for complex models, and for situations involving small samples. In addition, unlike the standard ML estimator, MCMC provides greater flexibility with respect to the distributions of the latent trait and the observed indicators. For example, the researcher who suspects that either set of variables (latent or observed) follows a skewed distribution can express this through the prior distributions set on each. Indeed, it is possible to use unique prior distributions for each indicator and each latent variable, thereby allowing the researcher to specifically tailor the analysis to the data at hand. Furthermore, if the prior and posterior distributions do not match (are not conjugate), the resulting posterior distribution is typically still unbiased (Kaplan, 2014). This robustness to the use of either non-informative or improper prior distributions means that the Bayesian estimator may prove particularly useful in contexts where the population latent and observed variables distributions are not known.

In order to understand the application of the Bayesian estimation approach to invariance testing, let us consider the 2 group case. We would like to assess whether factor loadings are equal between them. For each loading, a difference can be calculated between values for the groups. These differences are parameters to be estimated by the MCMC algorithm, such that each will have a prior and a posterior distribution. In the current study, a non-informative prior based on the normal distribution with a mean of 0 and a variance of 1,000 was used. For assessing invariance of a single factor loading, the 95% credibility interval of the posterior distribution of the difference in loadings between the groups can be used. When 0 falls within the interval invariance between the groups is said to hold, whereas if 0 falls outside of the interval then we conclude that the loading differs between the groups. If invariance assessment is carried out for multiple loadings, then some correction to the width of the interval may be desirable. It is important to note that alternative prior distributions to the one used in this explanation (and in the study) could be employed, with no loss of generality.

### Goals of the current study

The purpose of this simulation study was to compare the Type I error and power rates of multiple approaches to invariance assessment in the context of small samples and skewed latent traits, including MGCFA with standard ML estimation, ML estimation based on the skewed-*t* distribution, GSCA, and Bayesian estimation. More specifically, this study was designed to extend upon prior work on invariance assessment in the context of a skewed latent trait, and investigation of invariance testing with small sample sizes. Investigation of the combination of non-normal latent trait distributions and small sample sizes was a primary focus of the current study. Previous research with the skewed-*t* estimator has relied primarily on existing samples with 102 being the smallest. Given that the literature (e.g., Brown, 2016) suggests factor model parameter estimation using the standard maximum likelihood approach is more robust with larger sample sizes, coupled with the relative paucity of research focused on varying sample sizes for the skewed-*t* estimator, it was felt that the current study should focus on both latent trait distribution and sample size. Latent trait distributions included normal, and two levels of skewness, whereas sample sizes per group ranged from 25 to 1,000. Study details appear below.

Based upon work reviewed above, it was hypothesized that when a skewed latent trait appears in conjunction with a small sample size, the estimator based upon the skewed-*t* will provide the best control of the Type I error rate, followed by the Bayesian estimator. It is anticipated that the ML estimator will yield inflated Type I error rates in this combination of conditions. Insufficient prior research is available for hypotheses regarding the GSCA method to be developed. With respect to power, it is hypothesized that of the methods that were able to control the Type I error rate at the nominal level, the skewed-*t* and Bayesian estimators will yield comparable power levels. Again, there is insufficient prior research for hypotheses regarding the power of GSCA in this context. The third hypothesis is that for larger sample sizes the ML estimator will provide better control over the Type I error rate in the skewed latent trait conditions, though these rates will still exceed those of the skewed-*t* and Bayes estimators. Finally, there will be a positive relationship between inflated Type I error rates and degrees of skewness for all methods.

## Methodology

The research goals outlined above were addressed using a Monte Carlo simulation study with 1,000 replications per combination of study conditions. Data were generated from a single factor model conforming to Equation (1), with factor loadings for the individual indicator variables were set to 1, with the exception of cases when non-invariance was simulated, as is described below. The indicator variable error terms were generated from the *N* (0,1) distribution, and the intercepts were set to 0 for all indicators. The indicators themselves were generated from the *N* (0,1) distribution. Across all conditions, data for 2 groups was simulated. Non-invariance was simulated for factor loadings only (i.e., only MI was examined in the study). The conditions manipulated in this study were completely crossed, with the exception that the skewed-*t* estimator was not used with the normally distributed latent trait.

### Distribution of the latent trait

Three conditions were used for the distribution of the latent factor, including the normal, the skewed normal with skewness of 2, and the skewed normal with skewness of 4. These values were selected in order to provide a range of distributions for which the estimators could be assessed. The normal distribution was used in order to serve as a baseline, given that the ML estimator has been shown to control the Type I error rate and to yield high power in this case, when the sample size per group is 200 or more (French and Finch, 2006). The two skewed distribution conditions were used in order to assess the performance of the various methods when the ML assumption of a normally distributed latent trait has been violated, to both a mild (skewness = 2) and a more severe (skewness = 4) degree. The skewed-*t* estimator has been shown to be effective with skewed data in other modeling contexts, such as regression (Asparouhov and Muthén, 2016), but there has been little published work regarding its performance in the context of factor invariance assessment. The skewed variables were simulated using a method based upon that described in Azzalini and Capitanio (2014). The interested reader is referred to this monograph for details of how the method works.

### Number of indicator variables

Two conditions for the number of indicators were used: 10 and 20. These values were selected to reflect a short and long scale. Some conditions were simulated with 30 indicators, and the results were nearly identical to the 20 indicator case in terms of the outcome variables of Type I error and power rates. Given this similarity for 20 and 30 indicators, it was determined that results for both need not be included in the manuscript.

### Percent and magnitude of non-invariant loadings

The percentages of non-invariant indicators used in the study were 0, 10, 20, and 30%. Thus, for example, in the 10 indicator 20% non-invariant indicators condition 2 indicators were simulated to have different factor loadings between the groups, whereas for the 20 indicator 20% non-invariant indicators case, 4 variables were simulated to have different factor loadings. When non-invariance was simulated, the magnitude of loading differences was 0.5. Non-invariance was induced by simulating the group 1 loading for the non-invariant variable to be 1, and the group 2 loading to be 0.5.

### Sample size per group

The sample sizes per group were simulated to be 25, 50, 75, 100, 200, 500, and 1,000. These group sizes were simulated to range from what might be considered very small in practice (25 or 50) to large (500) and very large (1,000) per group. These sample size conditions were intended to test the methods in fairly extreme small sample size conditions, and in cases where prior work has demonstrated ML to work well.

### Estimation methods

The estimation methods used in the current study were ML under the assumption of normality, ML based on the skewed-*t* distribution, Bayes, and GSCA. The standard ML estimator was used in the study because it is typically the default for researchers conducting invariance analysis for continuous indicator variables. In addition, ML based on the skewed-*t* distribution was included in the study because it has been shown to provide accurate parameter estimates with skewed data (Asparouhov and Muthén, 2016). The Bayesian estimator was included because it has also been suggested for use when variables do not follow the normal distribution, and for small sample sizes (e.g., Muthén and Asparouhov, 2012; Kaplan, 2014). GSCA was included because prior work has demonstrated that it has positive potential for accurately fitting latent variable models with small sample sizes, nor does it rest on an assumption of normality (Hwang and Takane, 2004, 2010). Despite this positive literature, there has not been extensive prior work investigating the performance of GSCA in the context of invariance assessment, including with small samples and non-normal latent traits. Thus, the current study was designed to extend knowledge about the performance of this method under a broader array of conditions than has been investigated previously.

With regard to the Bayesian estimator, a Gibbs sampler was used, with a total of 50,000 samples after a burn-in period of 10,000 samples. The thinning rate was 10, yielding a posterior distribution of 5,000 data points. These estimation settings were used in the simulation study based upon initial work with combinations of conditions described above, for which parameter estimation convergence was achieved for exemplars of the conditions included in the study. Convergence rates were monitored for all simulation conditions. The prior distribution used for the factor loadings and intercepts was *N* (0, infinity), whereas the prior for the error variances was the inverse Gamma (−1,0).

Finally, the skewed-*t* estimator was not used in conjunction with the normally distributed latent trait, because in practice a researcher who assessed the distributional assumption of normality and found it to hold would be very unlikely to employ an estimator that is specifically designed for skewed latent traits. The other estimators are not so specifically tied to use with skewed data, and therefore were employed across distributional conditions.

### Study outcomes

There were two outcomes of interest: Type I error and power rates. Type I error was considered to be under control if it ranged between 0.025 and 0.075 (Bradley, 1978). In order to identify which manipulated terms, or interactions of these terms, were associated with Type I error and power rates, analysis of variance (ANOVA) was used. For each combination of conditions, the proportion of the 1,000 replications for which a statistically significant difference between the groups was calculated. These values were used as the dependent variable in the ANOVA, whereas the manipulated study factors and their interactions were the independent terms in the model (e.g., Harwell et al., 1996). A term was identified for further investigation if its ANOVA result was statistically significant, and the η^2^ effect size was 0.1 or greater. This value was used because it corresponds to the term accounting for at least 10% of the variance in Type I error or power rates. In addition to the primary outcomes of Type I error and power rates, the convergence rates of the methods were also examined.

## Results

### Convergence rates

**Figure 1:**
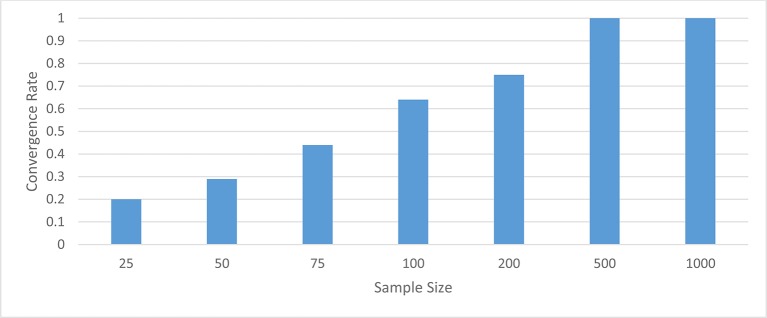
Convergence rates of MLE with Skewed-*t* distribution.

When the latent trait was normally distributed, the Bayesian, MLE, and GSCA estimators all had convergence rates of 100% for group sample sizes of 50 or more. When the group sample size was 25, Bayes and GSCA also had convergence rates of 100%. However, in this smallest sample size condition, MLE had a convergence rate of 72%. When the latent trait was skewed, convergence rates for the normal MLE, GSCA and Bayes were very similar to what was found for the normally distributed latent trait. Namely, for samples per group of 50 or more, convergence was 100% across conditions, and for 25 per group the normal MLE convergence rate was 67%, while remaining at 100% for Bayes and GSCA. Convergence rates for the skewed-*t* MLE estimator followed a very different pattern, however. Figure 1 displays the convergence rates for the skewed-*t* MLE by sample size per group. The convergence rate was below 100% except for group sample sizes of 500 and 1,000. For 200 subjects per group, the convergence rate of the skewed-*t* MLE was approximately 75%, and declined to 20% for 25 subjects per group.

### Normal latent trait distribution: Type I error rate

**Figure 2:**
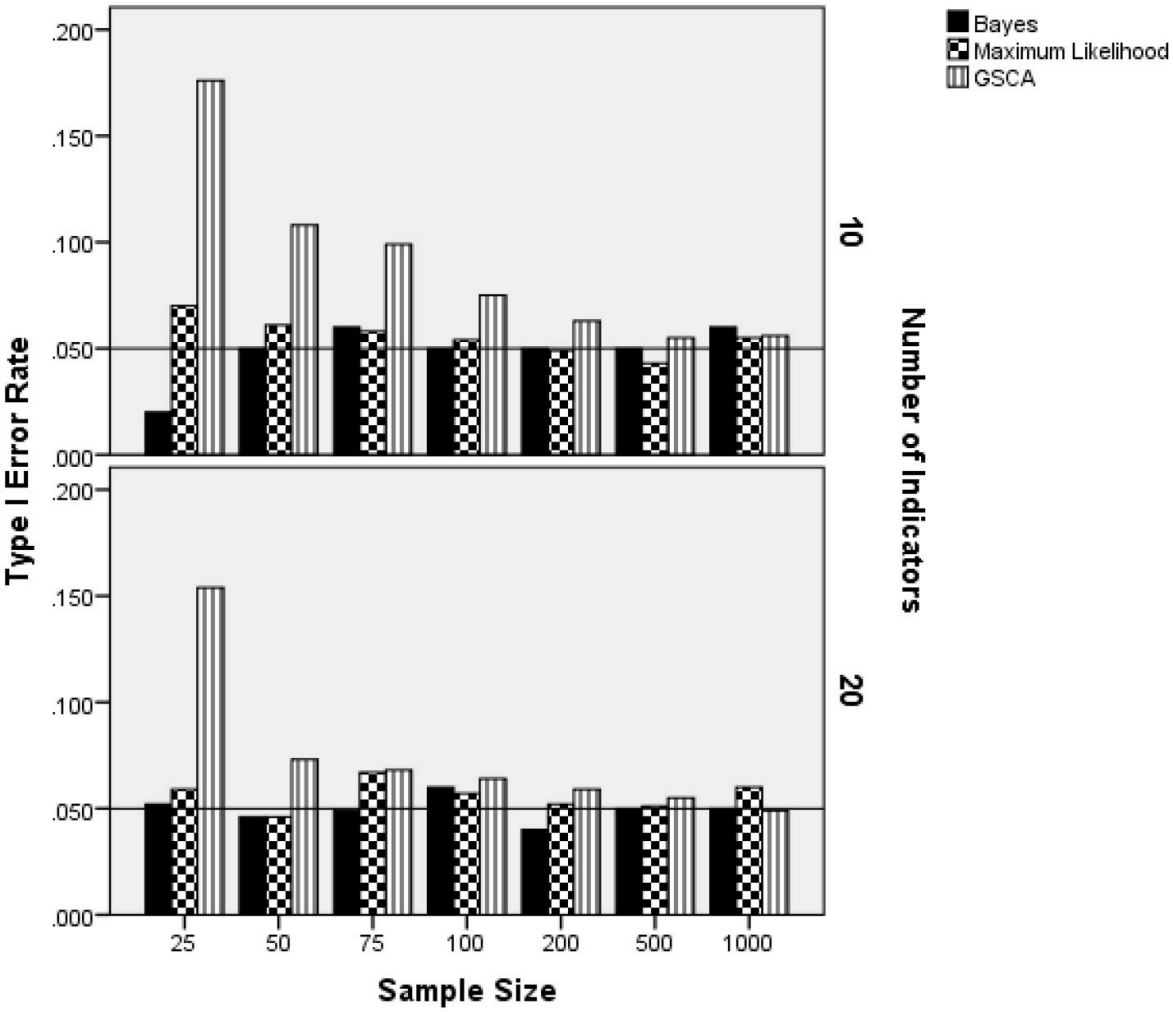
Type I error rate by sample size per group, number of indicators, and estimation method for normally distributed latent trait. Reference line at nominal Type I error rate of 0.05.

Because the skewed-*t* estimator was not used with the normally distributed latent trait condition, results for this distribution are analyzed and presented separately from the skewed distribution settings. The ANOVA identified the interaction of sample size per group by the number of indicators by the estimation method as being statistically significantly related to Type I error rate when the latent trait was normally distributed [*F*_(12, 8)_ = 3.767, *p* = 0.034, η^2^ = 0.85]. All other terms were either not statistically significant, were subsumed in this interaction, or did not have an effect size value in excess of 0.1. Figure 2 contains the Type I error rate by the sample size per group, the number of indicators, and estimation method. For 10 indicators, Type I error rates for ML were slightly elevated in the 25 per group sample size condition, but in control for the remainder of the conditions. The Bayes estimator yielded Type I error rates that were at the nominal 0.05 level for all sample size conditions, except for *N* = 25 per group, in which case it was 0.015. The GSCA Type I error rate in the 10 indicators condition was elevated above the 0.05 level for samples less than 200 per group. With regard to the 20 indicators condition, both the ML and Bayes estimators consistently maintained the nominal Type I error rate at 0.05. As in the 10 indicator condition, GSCA yielded Type I error rates above the nominal level for samples of 25 per group. However, for group sample sizes of 50 or more, the Type I error rate for the GSCA estimator never exceeded 0.07.

### Normal latent trait distribution: power

**Figure 3:**
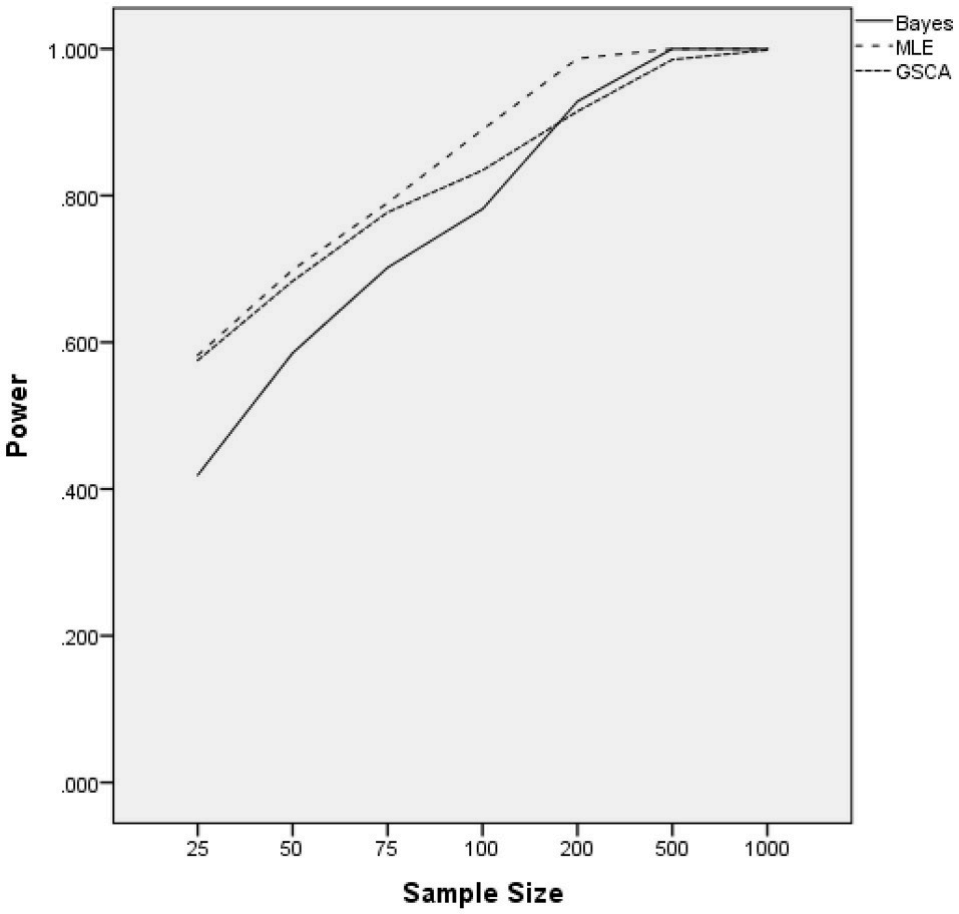
Power by estimation method and sample size for normally distributed latent trait.

**Figure 4:**
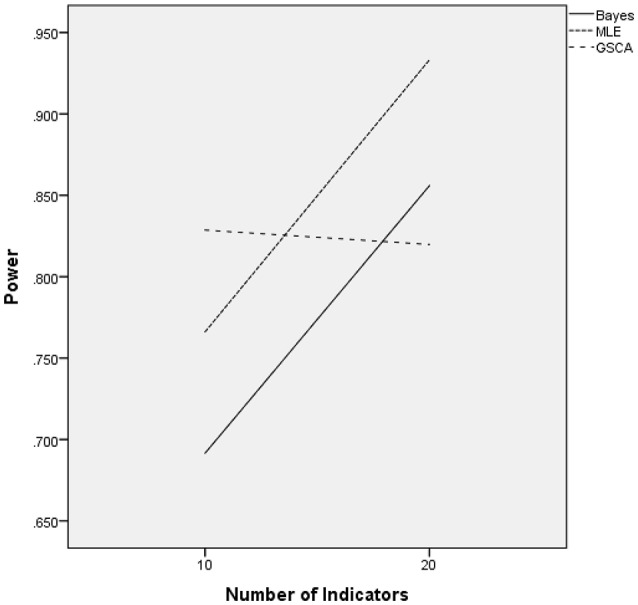
Power by estimation method and number of indicators for normally distributed latent trait.

The ANOVA for power as the outcome identified three terms as being statistically significant, including the interactions of estimation method by number of indicators [*F*_(2, 24)_ = 22.937, *p* = 0.001, η^2^ = 0.657], estimation method by sample size [*F*_(12, 24)_ = 27.024, *p* < 0.001, η^2^ = 0.932], and the main effect for percent of non-invariant loadings [*F*_(2, 12)_ = 4.618, *p* = 0.0033, η^2^ = 0.435]. Figure 3 displays the power rates by sample size and estimation method for the normally distributed latent trait condition. As anticipated, each of the methods yielded higher power rates for larger sample sizes per group. The Bayesian approach had the lowest power rates for samples of fewer than 200 per group, but exhibited comparable power to MLE and GSCA for group sizes of 200 or more. MLE had slightly higher power rates compared to GSCA for samples of 200 or fewer per group, whereas for samples of 500 and 1,000 power for the three methods was between 0.98 and 1.00. Figure 4 includes power rates for detecting non-invariance of factor loadings by estimation method and number of indicator variables. Power rates for both ML and the Bayesian estimator were higher for 20 indicators than for 10, whereas the power rates of GSCA were largely unaffected by the number of indicators. The mean power rates by the percent of non-invariant indicators appear in Table 1. As would be expected, power increased concomitantly with increases in the proportion of non-invariant indicators. Given the lack of a statistically significant interaction, it can be concluded that this relationship between the proportion of non-invariant indicators and power was equivalent across estimation methods.

**Table 1:** Power by proportion of non-invariant indicator variables.

**Proportion non-invariant (%)**	**Power**
10	0.627
20	0.755
30	0.979

### Skewed latent trait distribution: Type I error rate

**Figure 5:**
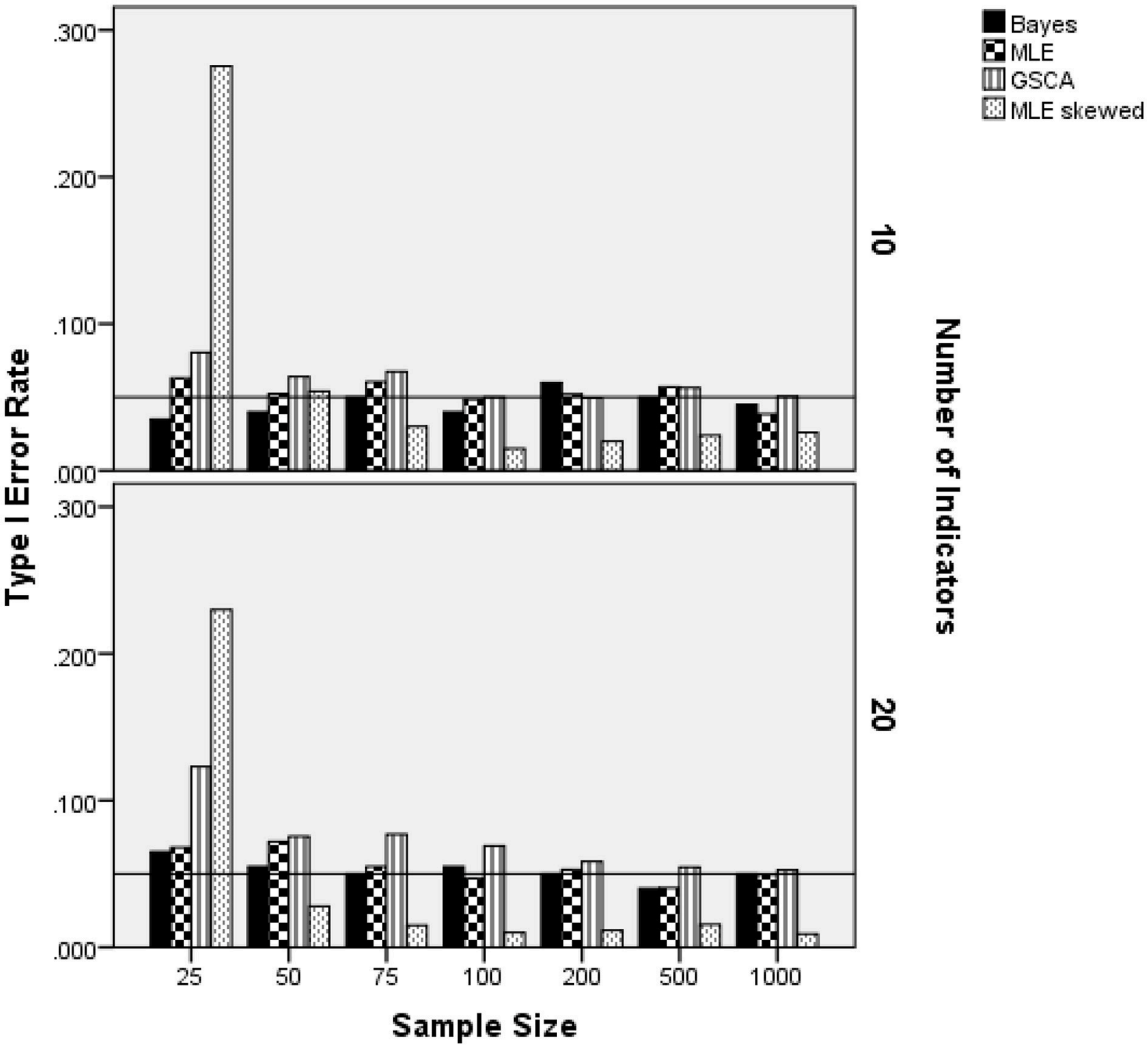
Type I error rate by estimation method, number of indicators, and sample size per group: skewed latent trait.

**Figure 6:**
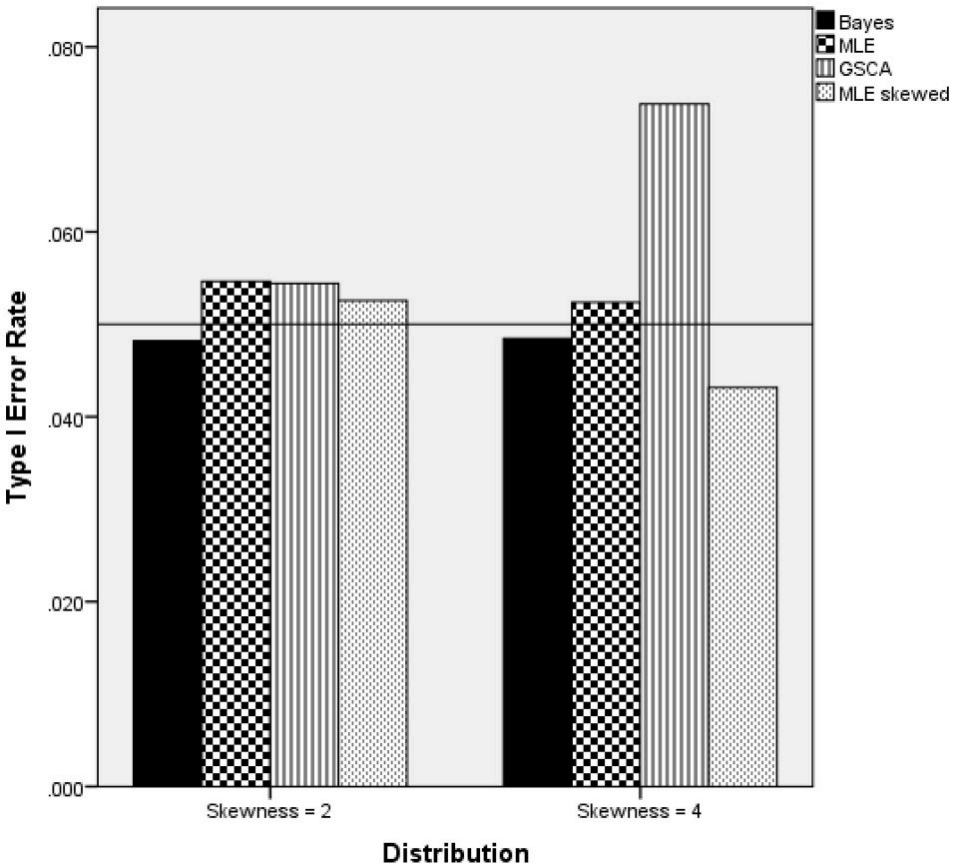
Type I error rate by estimation method and skewness of the latent trait.

Two interaction terms were statistically significantly related to the Type I error rate when the latent trait was skewed: estimation method by number of indicators by sample size per group [*F*_(18, 15)_ = 8.791, *p* = 0.007, η^2^ = 0.901], and estimation method by degree of skewness [*F*_(3, 15)_ = 18.307, *p* < 0.001, η^2^ = 0.948]. Figure 5 includes the Type I error rates by the estimation method, number of indicators, degree of skewness, and sample size per group. A reference line has been placed at the nominal 0.05 Type I error level. These results show that for the 25 per group sample size condition, the MLE based on the Skewed-*t* distribution had an elevated error rate for both 10 and 20 indicators. In addition, for the 20 indicators condition, the GSCA estimator also displayed an inflated Type I error rate. In all other combinations of estimation method, number of indicators, and sample size per group conditions the Type I error rate was under control when the latent trait was skewed. Figure 6 displays the Type I error rate by the estimation method and skewness of the latent trait. When the skewness was 2, all methods yielded Type I error rates that were in control, whereas for skewness of 4, GSCA yielded an inflated Type I error rate.

### Skewed latent trait distribution: power

**Figure 7:**
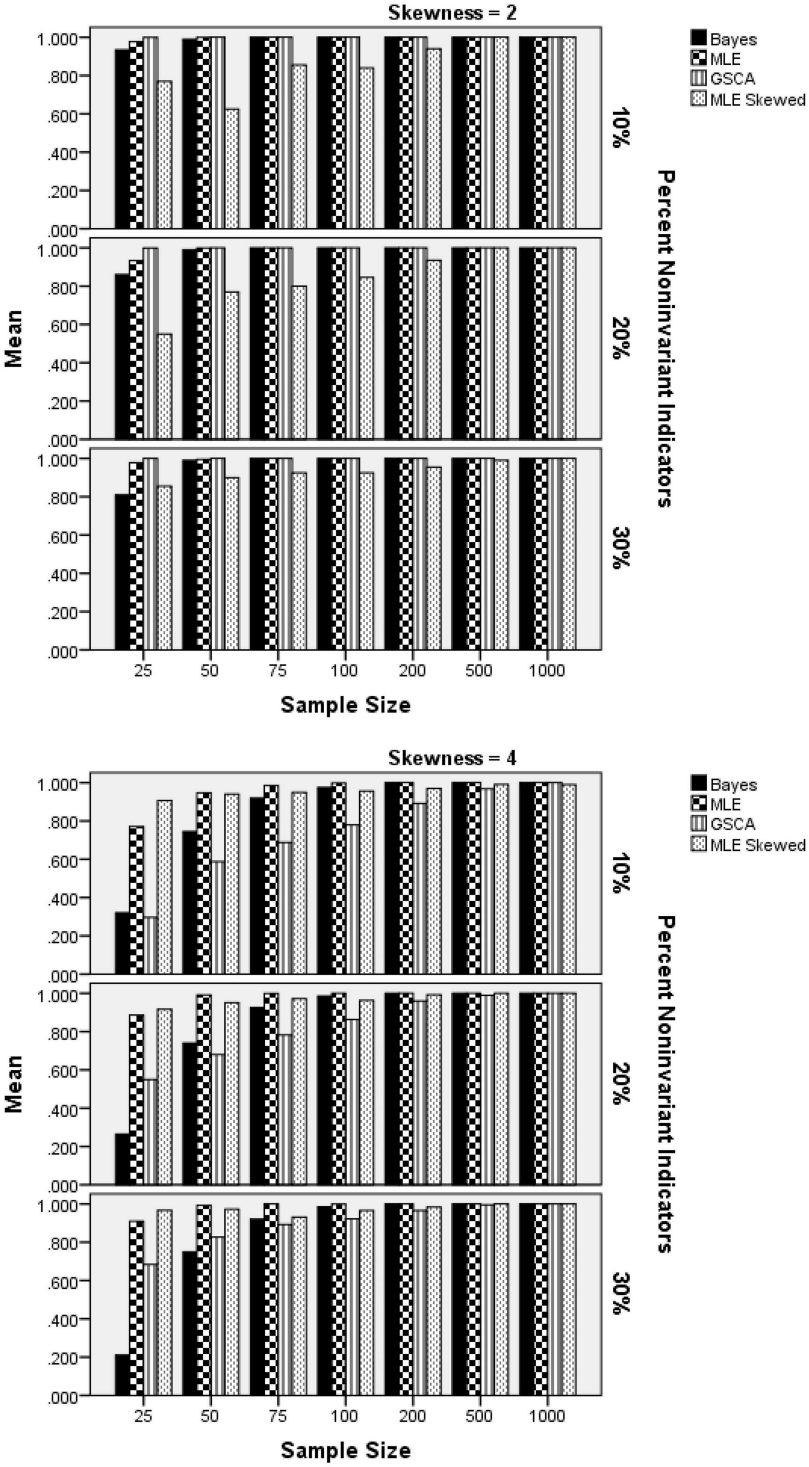
Power by estimation method, sample size per group, percent of non-invariant indicator variables, and latent trait skewness.

The ANOVA for the power rates for a skewed latent trait identified two statistically significant interactions, with η^2^ in excess of 0.1: estimation method by latent trait distribution by sample size per group, by percent of non-invariant indicators $\left[F_{\left(18,\;156\right)}=11.023,\;p<0.001,\;\eta^{2}=0.395\right]$, and estimation method by number of indicators [*F*_(3, 156)_ = 6.729, p < 0.001, η^2^ = 0.115]. Figure 7 contains power results by estimation method, sample size per group, percent of non-invariant indicators, and level of latent trait skewness. Panel 1 includes results for latent trait skewness of 2. Given the Type I error inflation for GSCA and the skewed-*t* estimator with a sample size of 25 per group, power results for this method with small samples must be interpreted with great caution. When skewness was 2, power for all methods except the MLE based on the skewed-*t* distribution was well above 0.8 across sample size conditions. For the smallest sample size per group, the Bayesian method displayed somewhat lower power than did MLE. For the more severe skewness level of 4, power for all estimation methods was generally lower than was the case for skewness of 2, when group sizes were 200 or less. In addition, power for the skewed-*t* MLE estimator was higher than that of the other methods for the smallest group size condition, and comparable to that of the normal based MLE across all other conditions. Power for the Bayesian estimator was substantially lower than that of either MLE approach for samples of 25 and 50, and power for GSCA was lower than that of the MLE estimators for samples of fewer than 200. Finally, when the latent trait skewness was 4, power increased for all methods concomitantly with increased in the percent of non-invariant indicator variables.

**Figure 8:**
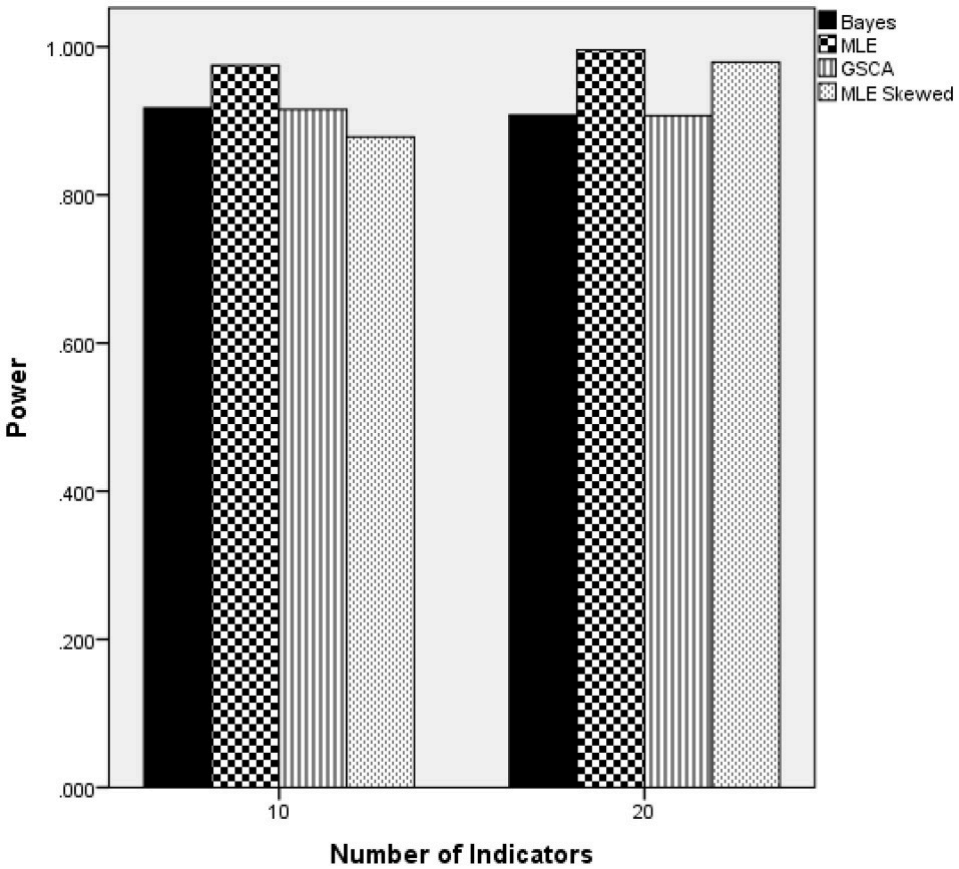
Power by estimation method by number of indicators: skewed latent trait.

Figure 8 includes power rates by estimation method and the number of indicator variables. These results show that the number of indicators was not related to power rates for the Bayesian, normal MLE, or GSCA estimators. However, the skewed-*t* MLE yielded higher power when there were 20 indicators, as opposed to 10. It is important to note that power was approximately 0.07 higher for the skewed-*t* MLE with 20 indicators. Furthermore, when there were 10 indicators the skewed-*t* estimator yielded the lowest power, whereas for 20 indicators its power was comparable to that of the normal based MLE, and higher than either Bayes or GSCA.

## Discussion

The purpose of this study was to compare the performance of MI assessment using several CFA estimation methods in conditions for which their performance might be questionable, namely with small samples and skewed latent traits. Conditions with larger samples and normally distributed latent traits were also included in the study to serve as points of comparison when the standard method using normal based ML and the chi-square difference test has been shown to work well. Based upon prior work, several hypotheses were posited regarding the performance of the various methods. The first hypothesis was that the skewed-t estimator would control the Type I error rate with a skewed latent trait and small sample size. In fact, the skewed-*t* estimator did control the Type I error rate for group sizes in excess of 25 per group. Contrary to our second hypothesis, however, normal based ML did not yield inflated Type I error rates. Next, we hypothesized that the skewed-*t* estimator would yield power comparable to that of the Bayesian approach when the latent trait was skewed. Results revealed that for the more severely skewed condition, the skewed-*t* estimator actually yielded higher power rates compared to Bayes, but that for the less skewed condition power for Bayes was higher compared to the skewed-*t*. In keeping with the third hypothesis, normal based ML was able to control the Type I error rate when the latent trait was skewed for larger sample size conditions; i.e., it was robust to non-normality with larger sample sizes. The last hypothesis, which stated a positive relationship between Type I error rates degrees of skewness was only supported for GSCA.

In summary, our results revealed that invariance testing based on the normal based ML estimator is generally very robust to sample size and latent trait distribution. This supports the robustness of ML under mild violations of assumptions. When the latent trait was normally distributed, ML maintained the nominal Type I error rate except for the case with 25 individuals per group, and 10 indicator variables. In addition, the normal based ML estimator yielded the highest power for correctly detecting a lack of MI, when the latent trait was normally distributed, across all sample size conditions. When the latent trait was skewed, ML was also able to control the Type I error rate at the nominal level, even when alternatives such as the skewed-*t* and GSCA estimators, could not for small sample sizes. With respect to power, the normal based ML performed well when compared to the other methods, yielding rates that were comparable to or greater than those of alternative methods.

From these results some implications for practice can be drawn. First, when the sample size per group is 50 or more, normal based ML is a useful estimator when the interest is in MI assessment, regardless of whether the latent trait is normally distributed or skewed to the extent that was simulated in this study. This approach largely maintained Type I error control, and yielded power rates that were comparable to or higher than those of the other methods. When the sample size per group was 25, ML had convergence rates of less than 100%, whereas GSCA and the Bayesian estimator maintained convergence rates of 100% across all sample size conditions. This could be a major advantage when working with low incidence populations or intervention studies with limited resources. In addition, unlike GSCA the Bayesian estimator maintained the nominal Type-I error rate across all conditions simulated in this study. On the other hand, the Bayesian estimator had much lower power at the smaller sample sizes than did the other approaches. Thus, a second implication for practice is that for sample sizes of 25 per group, researchers must make the choice from among 3 imperfect options. Perhaps first, the ML estimator should be tried, and if convergence is attained then the MI assessment results using this method are preferable, given its control over the Type I error rate and relatively high power. If convergence is not reached by the ML estimator, then the researcher must choose between using a method that will maintain the Type I error rate but at the cost of lower power (Bayes) or a method that has higher power but has an inflated Type I error rate (GSCA). Essentially, the researcher will need to decide whether the more serious error is to identify factor model parameter differences, in this case factor loadings, among groups when such differences are not present, or to miss finding such differences when they are present. Finally, invariance testing based on the skewed-*t* ML estimator must be more thoroughly explored and developed before it can be used in practice. Our results suggest that this estimation approach has very low convergence rates for small sample sizes, likely because of the additional parameters that must be estimated compared to the other methods. In addition, when convergence rates are reached, performance of this approach did not excel that of the normal based ML estimator. Thus, recommending this estimator for these conditions is not supported at this time. One last issue regarding invariance testing and small sample sizes should be addressed at this point. In practice, researchers engaging in such testing are typically expecting the hypothesized model to be invariant across groups. In other words, they are anticipating that the factor structure found to hold for one group will hold for another. Such invariance is important from an applied perspective as it is necessary in order for scores from the scale to be seen as carrying the same meaning for individuals from different subpopulations. Results of the current study indicate that the power for detecting a lack of invariance is fairly low for all methods when samples are very small (e.g., 25). Given the typical goal of invariance analyses to demonstrate that a given model structure holds across groups, this low power for small samples presents researchers with a potential conundrum, namely is a finding of statistical non-significant difference between groups the result of invariance holding, or of a lack of power for the procedures being used? The obvious answer to this conundrum is the development of invariance testing methods with relatively high power for small sample sizes. The results of this study would suggest that though some methods do exhibit more power than others when samples are small, there remains work to be done in developing approaches that have truly high power rates for very small samples.

### Directions for future research

We anticipate that these results aid understanding in the area of MI assessment when sample sizes are small and latent traits are skewed. Our results suggest several directions for future research. First, a wider array of skewness conditions should be simulated, and kurtosis should be manipulated to deepen the understanding of the performance of these estimation procedures under such situations. Second, more complex models should be examined. We only investigated performance of the methods using a simple 1-factor model to gain a baseline of results to inform and encourage future work. Thus, future work should consider MI assessment when multiple factors are present, and when structural elements relating latent variables are present in the model. In addition, work should assess the performance of these methods in testing for higher levels of model invariance, including for intercepts and random errors. It should also be noted that the current study examined performance of the various estimation techniques in the context of continuous indicator variables. Given that much invariance work is done with ordinal indicators in the form of items taken from scales, future simulation work should be conducted comparing the estimation methods studied here with categorical indicator models. Finally, with regard to the Bayesian estimator, alternatives to the non-informative priors for the difference in factor loadings that were used could be included to determine whether they have an impact on Type I error or power rates. Such investigations will continue to grow the understanding of how these estimation techniques paired with various model constraints, and sample and variable type combinations, influence the accuracy of invariance assessment. Such information can advance methodology in this area, and more importantly, inform practice to promote correct modeling conditions for given sets of variables and data. Of course, the aim is that this focus will help make the best inferences about individuals.

## Author contributions

All authors listed have made a substantial, direct and intellectual contribution to the work, and approved it for publication.

### Conflict of interest statement

The authors declare that the research was conducted in the absence of any commercial or financial relationships that could be construed as a potential conflict of interest.
